# Effect of Sex and Age on Physicochemical and Technological Characteristics in the *Longissimus thoracis et lumborum* Muscle in Botucatu Rabbits

**DOI:** 10.3390/ani15162368

**Published:** 2025-08-12

**Authors:** Erick Alonso Villegas-Cayllahua, Daniel Rodrigues Dutra, Ana Veronica Lino Dias, Érika Nayara Freire Cavalcanti, Nívea Maria Gomes Misson Carneiro, Leandro Dalcin Castilha, Hirasilva Borba

**Affiliations:** 1 Faculty of Agriculture and Veterinary Sciences, São Paulo State University, Jaboticabal 14884-900, Brazil; danielrdutra@hotmail.com (D.R.D.); ana.lino@unesp.br (A.V.L.D.); erikanayarac@gmail.com (É.N.F.C.); niveamariagomes@gmail.com (N.M.G.M.C.); hirasilva.borba@unesp.br (H.B.); 2 Department of Animal Science, State University of Maringá, Maringá 87020-900, Brazil; ldcastilha@uem.br

**Keywords:** chemical composition, loin, *Oryctolagus cuniculus*, rabbit farming

## Abstract

This study aimed to assess how sex and age influence the physicochemical and technological properties of the longissimus thoracis et lumborum muscle in Botucatu rabbits. Ten samples were used from each experimental group (females and males 3 and 12 months old). Color (L*, a*, and b*), pH, water-holding capacity, cooking weight loss, shear force, percentage of collagen (soluble, insoluble, and total), myofibrillar fragmentation index, sarcomere length, cholesterol concentrations, lipid oxidation, and chemical composition were evaluated. Sex influenced (*p* < 0.05) the chemical composition of the meat; young females presented meat with a higher concentration of lipids and cholesterol than males of the same age; however, the opposite results were obtained with the meat of 12-month-old rabbits. Age also influenced the meat’s characteristics: older rabbits had meat with more intense red and yellow colors and longer sarcomere length. In terms of chemical composition, 12-month-old rabbits had higher protein and lower soluble collagen and mineral matter compared to younger rabbits. Overall, age impacted the physicochemical properties, with older rabbits exhibiting more opaque, reddish meat, higher insoluble collagen, and lower moisture content. In addition, the influence of sex, especially on the chemical composition of meat in rabbits, must be emphasized.

## Simple Summary

This study evaluated how sex and age affect the physical, chemical, and technological characteristics of the *Longissimus thoracis et lumborum* muscle in Botucatu rabbits. Rabbits aged 6 and 12 months, both male and female, were analyzed. The parameters measured included color (L*, a*, and b*), pH, water-holding capacity, cooking loss, shear force, collagen content (soluble, insoluble, and total), the myofibrillar fragmentation index, sarcomere length, cholesterol levels, lipid oxidation, and overall chemical composition. Sex had a significant effect (*p* < 0.05) on meat composition. At 3 months, females had higher fat and cholesterol than males, while the opposite was observed at 12 months. Age also affected meat quality: older rabbits showed deeper red and yellow color tones, longer sarcomeres, more protein, and less soluble collagen and minerals. Their meat was more opaque, with more insoluble collagen and less moisture. In summary, age strongly influenced the meat’s physical and chemical traits, while sex mainly affected its composition. These findings highlight the importance of considering both age and sex when evaluating rabbit meat quality.

## 1. Introduction

Cuniculture has been expanding in recent times in several countries and reached a world production of 681,995.64 tons of meat in 2023 [1]. Among the main advantages of breeding rabbits are the characteristics of the species, such as the productive characteristics (rapid growth and rusticity) [2] and reproductive characteristics (precocity and prolificacy) [3], associated with the use of intensive breeding systems, which optimize the production of young rabbits and reduce the interval between births, since young rabbits wean in a shorter time, leading to a greater number of baby rabbits per year [4]. In addition to the search for rabbit strains that produce greater litter homogeneity—an attribute linked to higher reproductive efficiency—there is also a focus on improving the growth and productive performance of the kit [5,6].

However, in such intensive breeding systems, the productive life of females is increasingly shorter due to their greater use in the herd in the shortest possible time, leading to high replacement rates for breeding females and males that have completed their production cycle. In this context, replacement rates reaching as high as 120% may be observed [7] in order to maintain the herd’s productivity level, since as the female’s age increases, reproductive performance tends to decrease, with most replaced rabbits destined for slaughter along with young rabbits or, sometimes, sold as pets [8,9].

Such a situation represents a problem, since the literature shows that, in several species, the age of animal slaughter can influence the physical–chemical and technological characteristics of meat, and the meat of older animals can present undesirable characteristics for the consumer, such as less tenderness or redder meat [10,11].

While several studies have been conducted to assess the impact of age on rabbit meat quality [12,13,14], little information on the quality of meat from breeding rabbits is available.

Thus, this research focused on evaluating how sex and age affect the physicochemical and technological characteristics of the *Longissimus thoracis et lumborum* muscle in Botucatu breeding rabbits.

## 2. Materials and Methods

This research was conducted at the Laboratório de Análises de Alimentos de Origem Animal at the School of Agricultural and Veterinary Sciences, FCAV/UNESP, located in Jaboticabal, São Paulo, Brazil (21°08′ S, 48°11′ W, 583 m altitude), with approval from the institution’s Ethics Committee on the Use of Animals (CEUA) (protocol No. 5431/20).

### 2.1. Sample Collection and Experimental Procedure

Botucatu rabbits were used for the study and continuously mated and selected for disposal at 12 months of age, when they were slaughtered together with the F1 generation (at 3 months of age). The animals were raised in individual flat-deck cages (80 cm × 60 cm × 40 cm) in the Cuniculture Sector of the Faculty of Agricultural and Veterinary Sciences, FCAV/UNESP, Campus Jaboticabal, São Paulo, Brazil. The animals remained in the herd for eight months. The animals were raised under a 11.5 h light photoperiod, with only natural light being used during the experiment. No antibiotics were used during the experiment. The average temperature and humidity during the experiment were 25 °C and 68%, respectively. All rabbits received water ad libitum and a standard pellet diet labeled as “mixed diet,” which comprised 18% crude fiber, 15% mineral matter, 14% crude protein, 13% moisture, 10% calcium, 5% phosphorus, and 3% ether extract, tailored to their specific categories. Additionally, they had unlimited access to Coast-Cross grass hay (*Cynodon dactylon* (L.) pers.) available in their enclosures.

For the experiment, 40 samples of the *Longissimus thoracis et lumborum* muscle from randomly selected Botucatu rabbits were used. These animals were sourced from the same origin and were subjected to uniform handling conditions, organized into four distinct experimental groups (T1: young female rabbits at 3 months old; T2: female rabbits at 12 months old; T3: young male rabbits at 3 months old; and T4: male rabbits at 12 months old). Each treatment consisted of 10 rabbits, and the experimental unit was the rabbit itself. The rabbits were slaughtered in a commercial rabbit slaughterhouse inspected by the Federal Inspection Service (SIF).

After slaughter, the carcasses were kept in a cold chamber at 4 °C for 24 h before being placed in a freezing tunnel at −18 °C, with air circulation maintained at 3 m/s. After this process, the carcasses were transported in a temperature-regulated truck (−18 °C) to the Laboratório de Análises de Alimentos de Origem Animal (LaOra) in the Departamento de Biotecnologia Agropecuaria e Ambiental at FCAV/Unesp, Jaboticabal Campus, for subsequent physical and chemical assessments of meat quality.

Before being submitted to the analyses, the samples were thawed in a BOD (Biochemical Oxygen Demand) incubator (4 °C) (Eletrolab EL101/3 116 250W, Eletrolab, São Paulo, SP, Brazil) for 24 h so that the meat underwent a uniform thawing process, minimizing the risk of bacterial proliferation [15], and were subjected to the following physicochemical tests. After deboning, the *Longissimus thoracis et lumborum* samples were weighed, separated individually, and evaluated.

### 2.2. Physicochemical Analysis

#### 2.2.1. Meat Color

Meat color was assessed using a Minolta Chrome Meter model CR-400 colorimeter (Konica Minolta Sensing, Inc., Osaka, Japan) with the following settings: diffuse illumination/viewing angle 0, illuminant D65, specular component included, and an 8 mm aperture size. This colorimeter employs the CIELAB system [16], which incorporates three variables for color measurement: luminosity (L*), red intensity (a*), and yellow intensity (b*). Measurements were performed in triplicate on both surfaces of the *Longissimus thoracis et lumborum* muscle after a blooming time of 30 min, specifically on the external surface (the side of the muscle in contact with the rabbit’s skin) and the internal surface (the side in contact with the bone).

#### 2.2.2. pH, Water-Holding Capacity (WHC), Cooking Weight Loss (CWL), and Shear Force (SF)

The pH was determined in duplicate using a Testo digital parameter model 205 (Testo 205, Testo Inc., Sparta, NJ, USA) by directly inserting the electrode into the cranial region of the *Longissimus thoracis et lumborum* muscle. To calibrate the device, the electrode was first immersed in a buffer solution of pH 4.00 (Dinamica, Química Contemporânea Ltd.a., Indaiatuba, Brazil), followed by a buffer solution of pH 7.00 (Dinamica, Química Contemporânea Ltd.a., Indaiatuba, Brazil). The equipment automatically performs temperature compensation.

Water-holding capacity (WHC) was assessed using the method proposed by Hamm [17]. A 2 g portion of the *Longissimus thoracis et lumborum* muscle was measured and positioned between two sheets of filter paper and acrylic plates. The samples were then subjected to a 10 kg weight for five minutes to apply pressure. After the pressure duration, the acrylic plates were removed, and the sample was weighed again. The WHC was calculated using the formula below:

*WHC* (%) = (*Final weight* × 100)/*Initial weight*.

Cooking weight loss (CWL) was assessed following the methodology outlined by Honikel [18]. All samples of the *Longissimus thoracis et lumborum* muscle of approximate size and weight were weighed, packaged, and placed in a water bath at 85 °C for 45 min in a single cooking batch. After the cooked samples had cooled to room temperature, they were reweighed to determine the final weight, which was utilized to calculate the CWL, which was expressed as a percentage, as follows:

*CWL* (%) = (*Initial weight* − *Final weight*) × 100/*Initial weight*.

Cooked samples from the CWL analysis were utilized for shear force (SF) analysis. Once cooled to room temperature, the samples were divided into three sections, each approximately 1 cm^2^ in area. These sections were then cut using a Warner–Bratzler device connected to a Texture Analyser TA-XT2i texturometer (TAXT2i, Stable Micro Systems, Godalming, UK) equipped with a 50 kg load cell, ensuring that the fibers were oriented perpendicular to the cutting device. The force necessary to shear the samples was measured and reported in Newtons (N), in accordance with the methodology established by Lyon et al. [19].

#### 2.2.3. Percentage of Soluble, Insoluble, and Total Collagen

The three forms of collagen (soluble, insoluble, and total) were measured by quantifying the amino acid hydroxyproline in the samples. The analysis followed the methods outlined by Woessner Jr. [20] and Cross et al. [21], which were later adapted by Hadlich et al. [22] and subsequently by Carvalho et al. [23].

#### 2.2.4. Myofibrillar Fragmentation Index (MFI) and Sarcomere Length (SL)

The myofibrillar fragmentation index (MFI) was developed based on the approach described by Culler et al. [24] and supplemented by the biuret method [25], which is used to assess protein concentration in myofibril suspensions. The MFI is calculated using the formula MFI = optical density × 200.

The measurement of sarcomere length was conducted using the method established by Cross et al. [26]. The results are expressed in micrometers (µm).

#### 2.2.5. Chemical Composition

The analysis of the chemical composition was conducted following the guidelines set forth by the Association of Official Analytical Chemists [27] for evaluating moisture content (method 950.46), protein levels (method 977.14), and ash content (method 920.153). For the assessment of total lipid concentration, the procedure outlined by Bligh and Dyer [28] was employed.

#### 2.2.6. Total Cholesterol Concentrations and Lipid Oxidation

Total cholesterol levels were determined using the methodology proposed by Carvalho et al. [23].

Lipid oxidation was assessed using the thiobarbituric acid reactive substances (TBARS) test with the procedure outlined by Vyncke [29]. A 5 g portion of ground sample was extracted with trichloroacetic acid, and afterwards, a coloring reaction was initiated by heating with thiobarbituric acid. The measurements were performed at a wavelength of 532 nm using a spectrophotometer (Shimadzu UV-1800, Shimadzu Corporation, Kyoto, Japan). The results were presented as milligrams of malonaldehyde per kilogram of sample.

### 2.3. Statistical Analysis

A completely randomized experimental design was used in a 2 × 2 factorial scheme, composed of rabbits of two ages (3 and 12 months) vs. 2 sexes (females and males). The individual rabbit was considered as the experimental unit. The two independent variables, sex and age, were defined as fixed effects. The two main effects and their interactions were tested. The results were analyzed by the General Linear Models procedure of the Statistical Analysis System (SAS Institute Inc., Cary, NC, USA). The Shapiro–Wilk test was used to assess the normality of the results. The data were subjected to analysis of variance (ANOVA) and compared using the Tukey test, establishing a significance threshold at *p* < 0.05.

## 3. Results and Discussion

No interaction was detected (*p* > 0.05) between sex and age for luminosity (L*), red intensity (a*), or yellow intensity (b*) of the internal and external surfaces of the *Longissimus thoracis et lumborum* muscle (Table 1).

Luminosity was not influenced by sex or age (*p* > 0.05). In contrast, red intensity (a*) was significantly influenced by age (*p* < 0.05), with meat from 12-month-old rabbits appearing more reddish compared to meat from 3-month-old rabbits (*p* < 0.05). Similar results were described by Polak et al. [30], which may be related to higher concentrations of myoglobin in older rabbits. This is a compensatory mechanism of the organism, a product of decreased efficiency in gas exchange (O_2_-CO_2_) as the age of the animal increases, with myoglobin as the main protein that pigments the meat [30,31,32].

No interaction was detected (*p* > 0.05) between sex and age for pH, water-holding capacity (WHC), cooking weight loss (CWL), or shear force (SF) (Table 2).

pH, water retention capacity (WHC), cooking weight loss (CWL), and shear force (SF) were not influenced by sex and age of the rabbits (*p* > 0.05), which is relevant because these variables exert a great influence on the characteristics of meat quality that are relevant to the consumer [33,34,35].

No interaction was detected (*p* > 0.05) between sex and age on the percentage of soluble, insoluble, and total collagen (Table 3).

No effect (*p* > 0.05) was detected for sex on the percentages of soluble, insoluble, and total collagen. On the other hand, as the animal age increased, the percentages of soluble collagen decreased (*p* < 0.001) (0.10% and 0.04% in 3-month-old and 12-month-old rabbits, respectively) and the percentages of insoluble collagen in meat increased (*p* = 0.033) (0.13 and 0.17% in 3-month-old and 12-month-old rabbits, respectively). Possibly, this is because as the animal ages, the rate of collagen production reduces, giving time for the collagen cross-links that would normally be thermolabile (soluble collagen) to begin to stabilize and strengthen, making the generated collagen a permanently thermally stable protein (insoluble collagen); thus, higher temperatures are necessary to promote the tenderization of this meat [36,37,38].

Regardless of the differences in the percentages of insoluble and soluble collagen in meat as the animal matures, there are no changes in total collagen concentrations [35].

No interaction was detected (*p* > 0.05) between sex and age for the sarcomere length (Table 4).

Sex did not influence (*p* > 0.05) sarcomere length; however, regarding age, 12-month-old rabbits had meat with greater (*p* < 0.001) sarcomere length (2.20 µm) in relation to the 3-month-old rabbit meat (1.84 µm). Sarcomere lengths in 12-month-old rabbits exceeded 2 µm, suggesting that meat tenderness is likely not influenced by muscle structure denaturation (MFI) or collagen content [39,40]. Although MFI and collagen levels vary with age, this study found no significant differences in tenderness (*p* > 0.05) across age groups.

There was an interaction (*p* = 0.004) between sex and age for the myofibrillar fragmentation index (MFI) in the *Longissimus thoracis et lumborum* muscle of female and male Botucatu rabbits aged 3 and 12 months (Table 5).

The results of the analysis of the myofibrillar fragmentation index (MFI) in the *Longissimus thoracis et lumborum* muscle of Botucatu rabbits, as a function of sex and age, show significant differences in the combinations of these factors. Twelve-month-old females (101.11) had significantly higher MFI scores than three-month-old females (60, 90), indicating a clear age effect in this sex. When comparing 12-month-old females and males, no significant difference was observed, suggesting that the MFI score is similar between the sexes at this age. However, at three months, males had significantly higher MFI scores than females, indicating an early difference between the sexes. On the other hand, males did not show significant differences between three and twelve months, indicating that age does not affect the MFI score in this sex. However, it was observed that, independent of sex and age, the MFI values were greater than 60, which would be indicative of tender meat [21].

There was an interaction (*p* < 0.05) between sex and age for the chemical composition (moisture, lipids, protein, and mineral matter) of the *Longissimus thoracis et lumborum* muscle of female and male Botucatu rabbits at 3 and 12 months old (Table 6).

The chemical composition values obtained as noted in the present research are within the normal standards already described for rabbits [41].

The moisture analysis showed that, although there were no significant differences between males and females (*p* = 0.613) or between the 3- and 12-month-old rabbits (*p* = 0.212), the interaction between sex and age was highly significant (*p* < 0.001). The twelve-month-old females (71.15%) had a significantly higher moisture content than the three-month-old females (69.10%). Males, on the other hand, showed the opposite pattern: the three-month-olds (70.90%) had a higher moisture content than the 12-month-old males (69.68%). Furthermore, the 12-month-old females had significantly higher values than the 12-month-old males, while at three months, the males outperformed females. This reveals a significant interaction between sex and age in moisture content. As the age of males increased, the percentages of meat moisture decreased, which corroborates the literature [12,42], likely as a result of increased fat accumulation in the meat with the animal’s age, causing the concentration of the other components [12,43,44], with an inversely proportional relationship between the percentages of lipids and meat moisture [45,46].

In females, the lipid content decreased significantly with age, from 3.61% to 2.16% between 3 and 12 months, suggesting a marked reduction in fat accumulation. This trend was not observed in males (*p* > 0.05). At 3 months, females had significantly more lipids than males (3.61% vs. 2.47%), but at 12 months, the differences between sexes were no longer significant (2.16% vs. 1.99%). This indicates that females lost lipids more dramatically over time, generating a significant interaction (*p* = 0.019). The main effects of sex (*p* = 0.006) and age (*p* < 0.001) were also identified, reflecting that both factors influence lipid levels. However, the interaction between sex and age was also significant (*p* = 0.019), as females showed a greater reduction in lipid percentage with age compared to males. Thus, while in females the decrease was 1.45 percentage points, in males it was 0.48 percentage points, suggesting that the change in lipid composition is more pronounced in females. In the case of females, there was an increase (*p* < 0.05) in meat moisture percentages as age increased, which may be related to the decrease in lipid percentages in older females, as there is an inversely proportional relationship between moisture percentages and lipid percentages, since muscle has a limited volumetric capacity and due to the hydrophobic nature of fat, when one of these components tends to increase, the other tends to decrease. The reduction in lipid percentages with age may be attributed to the utilization of female energy reserves during pregnancy (especially during the last 10 days prior to giving birth) and lactation (in milk production), which leads to a decrease in lipid concentrations in meat [47,48]. The 3-month-old female rabbits had higher (*p* < 0.05) lipid concentrations (2.16%) in relation to the male rabbits (2.47%), possibly due to the action of sex hormones on fatty acid metabolism, such as estrogen, which influences lipid absorption and lipogenesis [49].

In males, protein content increased significantly with age, with 12-month-old males (23.46%) presenting more protein compared to 3-month-old males (21.75%), suggesting a greater accumulation of muscle or structural proteins in older animals. In contrast, with respect to females, there were no significant differences between 3- and 12-month-olds (22.45% and 22.00%, respectively). At 3 months of age, there were no significant differences between sexes (22.45% in females and 21.75% in males), but at 12 months, males significantly outnumbered females (23.46% vs. 22.00%). This divergence in the growth response between males and females resulted in a significant interaction between age and sex (*p* = 0.009). This difference in trends between males and females in relation to age highlights that the effect of age on protein varies by sex: in females, protein content decreased slightly with age, while in males it increased significantly, which aligns with the results obtained by Gondret et al. [43] probably as a result of higher protein accumulation in the meat as the animal matures.

Females experienced a significant decrease in mineral content with aging, dropping from 1.68% to 1.17% between 3 and 12 months. In contrast, males maintained a relatively stable mineral content with age (1.37% to 1.40%), with no significant differences between ages. At 3 months, females had significantly more mineral content than males (1.68% vs. 1.37%), which was also observed by Gašperlin et al. [13], while at 12 months this relationship was reversed, with males showing slightly more mineral content than females (1.40% vs. 1.17%). In terms of age, females experienced a significant decrease in their mineral matter percentage from 1.68% at 3 months to 1.17% at 12 months, corroborating Polak et al. [30], while in males, the percentage remained stable, with 1.37% at 3 months and 1.40% at 12 months. The interaction between sex and age was significant (*p* = 0.010), indicating that the change in mineral matter content with age depends on sex: in females, there was a substantial decrease, while in males there was no appreciable change.

No interaction was detected (*p* > 0.05) between sex and age regarding lipid oxidation (Table 7).

There was no effect of sex (*p* > 0.05). On the other hand, a significant effect was present for animal age. Meat from the 12-month-old rabbits showed lower (*p* < 0.05) oxidation values (0.32 mg MDA/kg) in relation to the 3-month-old rabbits (0.90 mg MDA/kg).

This is possibly related to changes in the lipid profile of rabbits in relation to age, because as the age of the animal increases, there is often a decrease in the concentrations of polyunsaturated fatty acids (PUFAs) and an increase in the concentrations of saturated fatty acids (SFAs) in the meat. PUFAs are the most susceptible acids to lipid oxidation processes due to their molecular structure, as they have double bonds, which cause their weakening. Thus, meat from older animals that would normally have higher concentrations of SFA compared to meat from young animals would be less susceptible to lipid oxidation processes [50,51].

An interaction (*p* < 0.004) was detected between sex and age of female rabbits for the cholesterol concentration of the *Longissimus thoracis et lumborum* muscle of female and male Botucatu rabbits of 3 and 12 months old (Table 8).

The meat of females showed higher (*p* < 0.05) cholesterol concentrations compared to the meat of 3-month-old male rabbits; however, this trend was reversed for cholesterol concentrations in the 12-month-old rabbits. Nevertheless, independent of sex and age, the cholesterol data shown in the experiment are within the standards indicated in the literature (45 to 85 mg/100 g) [13,52].

## 4. Conclusions

We concluded that age influences the physicochemical characteristics of rabbit meat. The 12-month-old rabbits presented meat with a more reddish color, a muscle structure with higher percentages of insoluble collagen, longer sarcomere lengths, higher percentages of protein and fat, and lower percentages of moisture compared to the meat from the 3-month-old rabbits. In addition, the influence of sex especially on the chemical composition of meat in rabbits has to be emphasized.

## Figures and Tables

**Table 1:** Means (±SEM) of luminosity (L*), red intensity (a*), and yellow intensity (b*) of the external and internal surface of the *Longissimus thoracis et lumborum* muscle of female and male Botucatu rabbits of 3 and 12 months old.

Variable		*Animal Sex (S)*		*Animal Age (A)*		*p-Value*		
		Female	Male	3 Months	12 Months	*P* (S)	*P* (A)	*P* (S × A)
External surface	L*	53.4 ± 0.7	53.3 ± 0.8	54.4 ± 0.7	52.3 ± 0.8	0.963	0.065	0.430
	a*	6.0 ± 0.4	5.3 ± 0.4	4.7 ± 0.4 ^B^	6.6 ± 0.4 ^A^	0.300	0.004	0.392
	b*	1.8 ± 0.4	2.2 ± 0.4	1.2 ± 0.4 ^B^	2.8 ± 0.4 ^A^	0.499	0.012	0.778
Internal surface	L*	54.4 ± 0.8	52.6 ± 0.9	53.3 ± 0.8	53.8 ± 0.5	0.128	0.718	0.512
	a*	4.4 ± 0.5	5.9 ± 0.5	4.8 ± 0.5 ^B^	5.6 ± 0.5 ^A^	0.443	0.025	0.218
	b*	0.2 ± 0.2	0.2 ± 0.3	0.1 ± 0.2	0.3 ± 0.3	0.905	0.504	0.283

^A,B^ Means accompanied by different letters (in the lines) indicate significant differences as determined by Tukey’s test (*p* < 0.05). SEM: standard error of the mean; S: animal sex; A: animal age; S × A: interaction of sex and age of the animal.

**Table 2:** Means (±SEM) of pH values, water-holding capacity (WHC), cooking weight loss (CWL), and shear force (SF) of the *Longissimus thoracis et lumborum* muscle of female and male Botucatu rabbits at 3 and 12 months old.

Variable	*Animal Sex (S)*		*Animal Age (A)*		*p-Value*		
	Female	Male	3 Months	12 Months	*P* (S)	*P* (A)	*P* (S × A)
pH	5.87 ± 0.04	5.97 ± 0.04	5.91 ± 0.04	5.92 ± 0.04	0.163	0.891	0.203
WHC (%)	71.92 ± 0.84	72.87 ± 0.91	72.70 ± 0.82	72.09 ± 0.93	0.451	0.632	0.616
CWL (%)	23.33 ± 0.77	24.05 ± 0.83	24.58 ± 0.75	22.80 ± 0.84	0.533	0.126	0.146
SF (N)	13.05 ± 0.88	13.54 ± 0.88	13.64 ± 0.79	13.05 ± 0.98	0.708	0.645	0.485

SEM: standard error of the mean; S: animal sex; A: animal age; S × A: interaction of sex and age of the animal.

**Table 3:** Means (±SEM) of percentage values of soluble, insoluble, and total collagen of the *Longissimus thoracis et lumborum* muscle of female and male Botucatu rabbits at 3 and 12 months old.

Variable	*Animal Sex (S)*		*Animal Age (A)*		*p-Value*		
	Female	Male	3 Months	12 Months	*P* (S)	*P* (A)	*P* (S × A)
Soluble collagen (%)	0.07 ± 0.01	0.08 ± 0.01	0.10 ± 0.01 ^A^	0.05 ± 0.01 ^B^	0.461	<0.001	0.140
Insoluble collagen (%)	0.14 ± 0.01	0.16 ± 0.01	0.13 ± 0.01 ^B^	0.17 ± 0.01 ^A^	0.409	0.033	0.351
Total collagen (%)	0.22 ± 0.01	0.24 ± 0.01	0.24 ± 0.01	0.21 ± 0.01	0.443	0.256	0.218

^A,B^ Means accompanied by different letters (in the lines) indicate significant differences as determined by Tukey’s test (*p* < 0.05). SEM: standard error of the mean; S: animal sex; A: animal age; S × A: interaction of sex and age of the animal.

**Table 4:** Means (±SEM) of sarcomere length (SL) values of the *Longissimus thoracis et lumborum* muscle of female and male Botucatu rabbits at 3 and 12 months old.

Variable	*Animal Sex (S)*		*Animal Age (A)*		*p-Value*		
	Female	Male	3 Months	12 Months	*P* (S)	*P* (A)	*P* (S × A)
SL (μm)	1.92 ± 0.02	1.95 ± 0.02	1.84 ± 0.02 ^B^	2.20 ± 0.02 ^A^	0.427	<0.001	0.550

^A,B^ Means accompanied by different letters (in the lines) indicate significant differences as determined by Tukey’s test (*p* < 0.05). SEM: standard error of the mean; S: animal sex; A: animal age; S × A: interaction of sex and age of the animal.

**Table 5:** Means (±SEM) of sex and age interaction for myofibrillar fragmentation index (MFI) of the *Longissimus thoracis et lumborum* muscle of female and male Botucatu rabbits at 3 and 12 months old.

*Animal Sex (S)*	*Animal Age (A)*		*p-Value*		
	3 Months	12 Months			
MFI			P (S)	P (A)	P (S × A)
**Female**	60.90 ± 5.91 ^Bb^	101.11 ± 5.91 ^Aa^	0.249	0.002	0.004
**Male**	87.31 ± 5.91 ^Aa^	89.31 ± 7.07 ^Aa^			

^A,B, a,b^ Means accompanied by different letters in columns (uppercase) and in lines (lowercase) indicate significant differences as determined by Tukey’s test (*p* < 0.05). SEM: standard error of the mean; S: animal sex; A: animal age; S × A: interaction of sex and age of the animal.

**Table 6:** Means (±SEM) of values for the interaction of sex and age for the percentage of moisture, lipids, protein, and mineral matter of the *Longissimus thoracis et lumborum* muscle of female and male Botucatu rabbits at 3 and 12 months old.

*Animal Sex (S)*	*Animal Age (A)*		*p-Value*		
	3 Months	12 Months			
**Moisture (%)**			***P* (S)**	***P* (A)**	***P* (S × A)**
**Female**	69.10 ± 0.30 ^Bb^	71.15 ± 0.32 ^Aa^	0.613	0.212	<0.001
**Male**	70.90 ± 0.30 ^Aa^	69.68 ± 0.36 ^Bb^			
Lipid (%)			*P* (S)	*P* (A)	*P* (S × A)
**Female**	3.61 ± 0.31 ^Aa^	2.16 ± 0.31 ^Ab^	0.006	<0.001	0.019
**Male**	2.47 ± 0.33 ^Ba^	1.99 ± 0.36 ^Aa^			
Protein (%)			*P* (S)	*P* (A)	*P* (S × A)
**Female**	22.45 ± 0.36 ^Aa^	22.00 ± 0.38 ^Ba^	0.882	0.113	0.009
**Male**	21.75 ± 0.36 ^Ab^	23.46 ± 0.43 ^Aa^			
Mineral matter (%)			*P* (S)	*P* (A)	*P* (S × A)
**Female**	1.68 ± 0.09 ^Aa^	1.17 ± 0.09 ^Ab^	0.021	0.636	0.010
**Male**	1.37 ± 0.08 ^Ba^	1.40 ± 0.10 ^Aa^			

^A,B, a,b^ Means accompanied by different letters in columns (uppercase) and in lines (lowercase) indicate significant differences as determined by Tukey’s test (*p* < 0.05). SEM: standard error of the mean; S: animal sex; A: animal age; S × A: interaction of sex and age of the animal.

**Table 7:** Means (±SEM) of lipid oxidation values (TBARS) of the *Longissimus thoracis et lumborum* muscle of female and male Botucatu rabbits at 3 and 12 months old.

Variable	*Animal Sex (S)*		*Animal Age (A)*		*p-Value*		
	Female	Male	3 Months	12 Months	*P* (S)	*P* (A)	*P* (S × A)
TBARS (mg MDA/kg)	0.59 ± 0.08	0.63 ± 0.09	0.90 ± 0.08 ^A^	0.32 ± 0.09 ^B^	0.762	<0.001	0.902

^A,B^ Means accompanied by different letters (in the lines) indicate significant differences as determined by Tukey’s test (*p* < 0.05). SEM: standard error of the mean; S: animal sex; A: animal age; S × A: interaction of sex and age of the animal.

**Table 8:** Means (±SEM) of the values for interaction of sex and age for the cholesterol concentration of the *Longissimus thoracis et lumborum* muscle of female and male Botucatu rabbits at 3 and 12 months.

*Animal Sex (S)*	*Animal Age (A)*		*p-Value*		
	3 Months	12 Months			
**Cholesterol (mg/100 g)**			***P* (S)**	***P* (A)**	***P* (S × A)**
Female	77.80 ± 1.86 ^Aa^	71.55 ± 1.73 ^Bb^	0.249	0.002	0.004
Male	72.16 ± 1.86 ^Bb^	79.20 ± 1.98 ^Aa^			

^A,B, a,b^ Means accompanied by different letters in columns (uppercase) and in lines (lowercase) indicate significant differences as determined by Tukey’s test (*p* < 0.05). SEM: standard error of the mean; S: animal sex; A: animal age; S × A: interaction of sex and age of the animal.

## Data Availability

The data that support the findings will be available in Repositorio Institucional da UNESP at https://hdl.handle.net/11449/251659 (accessed on 10 May 2025) following an embargo from the date of publication to allow time for the article to be published first.
