# Correction: Villegas-Cayllahua et al. Effect of Sex and Age on Physicochemical and Technological Characteristics in the *Longissimus thoracis et lumborum* Muscle in Botucatu Rabbits. *Animals* 2025, *15*, 2368

**DOI:** 10.3390/ani16050694

**Published:** 2026-02-24

**Authors:** Erick Alonso Villegas-Cayllahua, Daniel Rodrigues Dutra, Ana Veronica Lino Dias, Érika Nayara Freire Cavalcanti, Nívea Maria Gomes Misson Carneiro, Leandro Dalcin Castilha, Hirasilva Borba

**Affiliations:** 1 Faculty of Agriculture and Veterinary Sciences, São Paulo State University, Jaboticabal 14884-900, Brazil; danielrdutra@hotmail.com (D.R.D.); ana.lino@unesp.br (A.V.L.D.); erikanayarac@gmail.com (É.N.F.C.); niveamariagomes@gmail.com (N.M.G.M.C.); hirasilva.borba@unesp.br (H.B.); 2 Department of Animal Science, State University of Maringá, Maringá 87020-900, Brazil; ldcastilha@uem.br

**Missing Funding**

In the original publication [1], the funder Coordenação de Aperfeiçoamento de Pessoal de Nível Superior-Brasil (CAPES)-Finance Code 001 to Erick Alonso Villegas Cayllahua was not included. 

**Text Correction**

There was an error in the original publication, in the Simple Summary section, the sentence currently reads:

“At 6 months, females had higher fat and cholesterol than males, while the opposite was observed at 12 months”.

A correction has been made and should read as follows:

“At 3 months, females had higher fat and cholesterol than males, while the opposite was observed at 12 months”.

The authors state that the scientific conclusions are unaffected. This correction was approved by the Academic Editor. The original publication has also been updated.

## References

[1] Villegas-Cayllahua E.A., Dutra D.R., Dias A.V.L., Cavalcanti É.N.F., Carneiro N.M.G.M., Castilha L.D., Borba H. (2025). Effect of Sex and Age on Physicochemical and Technological Characteristics in the *Longissimus thoracis et lumborum* Muscle in Botucatu Rabbits. Animals.

